# Diagnostic utility of a novel magnifying endoscopic classification system for superficial Barrett’s esophagus-related neoplasms: a nationwide multicenter study

**DOI:** 10.1007/s10388-021-00841-1

**Published:** 2021-05-30

**Authors:** Kenichi Goda, Manabu Takeuchi, Ryu Ishihara, Junko Fujisaki, Akiko Takahashi, Yasuhiro Takaki, Dai Hirasawa, Kumiko Momma, Yuji Amano, Kazuyoshi Yagi, Hiroto Furuhashi, Satoru Hashimoto, Takashi Kanesaka, Tomoki Shimizu, Yoichiro Ono, Taku Yamagata, Junko Fujiwara, Takane Azumi, Gen Watanabe, Yasuo Ohkura, Masako Nishikawa, Tsuneo Oyama

**Affiliations:** 1 Gastrointestinal Endoscopy Center, Dokkyo Medical University, Tochigi, 321-0293 Japan; 2 Department of Gastroenterology, Nagaoka Red Cross Hospital, Niigata, Japan; 3 Department of Gastrointestinal Oncology, Osaka International Cancer Institute, Osaka, Japan; 4 Department of Gastroenterology, Japanese Foundation for Cancer Research, Cancer Institute Hospital, Tokyo, Japan; 5 Department of Endoscopy, Saku Central Hospital Advanced Care Center, Nagano, Japan; 6 Department of Gastroenterology, Ashiya Central Hospital, Fukuoka, Japan; 7 Department of Gastroenterology, Sendai Kousei Hospital, Miyagi, Japan; 8 Department of Endoscopy, Tokyo Metropolitan Cancer and Infectious Disease Center Komagome Hospital, Tokyo, Japan; 9 Department of Endoscopy, New Tokyo Hospital, Chiba, Japan; 10 Department of Gastroenterology and Hepatology, Uonuma, Institute of Community Medicine, Niigata University, Medical and Dental Hospital, Niigata, Japan; 11 Department of Endoscopy, The Jikei University School of Medicine, Tokyo, Japan; 12 Division of Gastroenterology and Hepatology, Graduate School of Medical and Dental Sciences, Niigata University, Niigata, Japan; 13 Department of Gastroenterology, Fukuoka University Chikushi Hospital, Chikushino, Japan; 14 Department of Gastroenterology, Sendai City Medical Center, Miyagi, Japan; 15 Department of Gastroenterology, International University of Health and Welfare Ichikawa Hospital, Chiba, Japan; 16 Department of Pathology, Niigata Cancer Center Hospital, Niigata, Japan; 17 Pathology and Cytology Center, PCL Japan, Saitama, Japan; 18 Clinical Research Support Center, The Jikei University School of Medicine, Tokyo, Japan

**Keywords:** Magnification endoscopy, Classification system, Barrett’s esophagus, Barrett's esophagus-related neoplasia, Esophageal adenocarcinoma

## Abstract

**Background:**

Currently, no classification system using magnification endoscopy for the diagnosis of superficial Barrett’s esophagus (BE)-related neoplasia has been widely accepted. This nationwide multicenter study aimed to validate the diagnostic accuracy and reproducibility of the magnification endoscopy classification system, including the diagnostic flowchart developed by the Japan Esophageal Society—Barrett’s esophagus working group (JES-BE) for superficial Barrett’s esophagus-related neoplasms.

**Methods:**

The JES-BE acquired high-definition magnification narrow-band imaging (HM-NBI) images of non-dysplastic and dysplastic BE from 10 domestic institutions. A total of 186 high-quality HM-NBI images were selected. Thirty images were used for the training phase and 156 for the validation (test) phase. We invited five non-experts and five expert reviewers. In the training phase, the reviewers discussed how to correctly predict the histology based on the JES-BE criteria. In the validation phase, they evaluated whether the criteria accurately predicted the histology results according to the diagnostic flowchart. The validation phase was performed immediately after the training phase and at 6 weeks thereafter.

**Results:**

The sensitivity and specificity for all reviewers were 87% and 97%, respectively. Overall accuracy, positive predictive value, and negative predictive value were 91%, 98%, and 83%, respectively. The overall strength of inter-observer and intra-observer agreements for dysplastic histology prediction was *κ* = 0.77 and *κ* = 0.83, respectively. No significant difference in diagnostic accuracy and reproducibility between experts and non-experts was found.

**Conclusion:**

The JES-BE classification system, including the diagnostic flowchart for predicting dysplastic BE, is acceptable and reliable, regardless of the clinician’s experience level.

## Introduction

Barrett’s esophagus (BE) is the primary precursor of dysplasia and esophageal adenocarcinoma (EAC), which are referred to as BE-related neoplasms. EAC is one of the most rapidly increasing cancers in the West. Recently, the incidence of EAC has slightly increased in the East, including Japan [1, 2]. The 5-year overall survival of patients with EAC, including advanced-stage cancer, has been reported to be poor (15–25%) [3]. However, studies showed an excellent prognosis among patients with superficial BE-related neoplasms (SBERN), including dysplasia and EAC confined to the submucosal layer [4, 5]. Early detection is the primary key factor for a favorable prognosis among patients with EAC.

SBERN, especially the flat macroscopic type, is often difficult to detect by standard white-light endoscopy [6]. Current guidelines in the West recommend endoscopic surveillance for patients with BE using random four-quadrant biopsies, in which samples are obtained based on the Seattle protocol to detect SBERN. However, the Seattle protocol is labor-intensive, time-consuming, expensive, and associated with a high risk of sampling error [7, 8].

Numerous studies have explored a directed biopsy method to overcome the shortcomings of the Seattle protocol [9]. A recent meta-analysis suggested that acetic acid enhancement, narrow-band imaging (NBI), and endoscopy-based confocal laser endomicroscopy are promising techniques for targeted biopsy that could be employed to eliminate the need for random biopsies [10]. Several classifications using NBI magnification endoscopy have been proposed, thereby suggesting the high utility of the classifications for the diagnosis of SBERN [11–14]. However, none of them have been widely accepted; thus, recent studies attempted to integrate and simplify the magnifying endoscopic classifications [15–17].

**Fig. 1:**
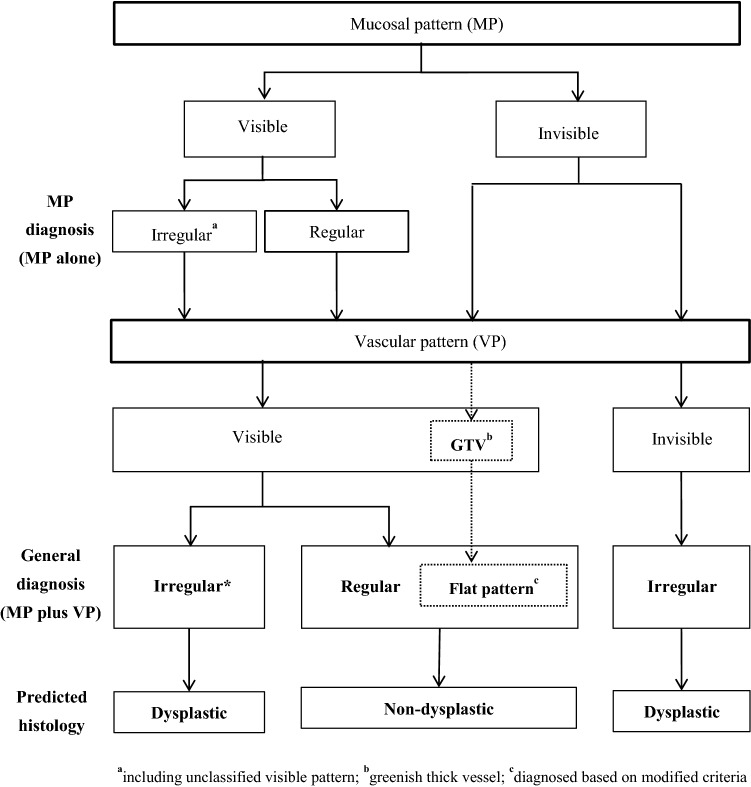
Diagnostic flowchart of the JES-BE classification system

We, the Japan Esophageal Society—Barrett’s esophagus working group (JES-BE), have developed and proposed a new magnifying endoscopic classification system, including a diagnostic flowchart (Fig. 1), to identify SBERN, namely, the JES-BE classification [18]. The classification system incorporates well-known diagnostic criteria for early gastric cancer and modified criteria for a flat pattern, with a goal of wide acceptance among not only experts but non-experts [19–22]. This nationwide multicenter study aimed to validate the diagnostic accuracy and reproducibility of the JES-BE classification for SBERN.

## Methods

### Working group and development of consensus-based classification system

The JES created a working group composed of 11 expert gastrointestinal endoscopists and two pathologists with expertise in gastrointestinal neoplasms. The working group developed the JES-BE classification based on consensus among the 13 working committee members, as reported previously [18].

### Diagnostic flowchart of JES-BE classification system

**Fig. 2:**
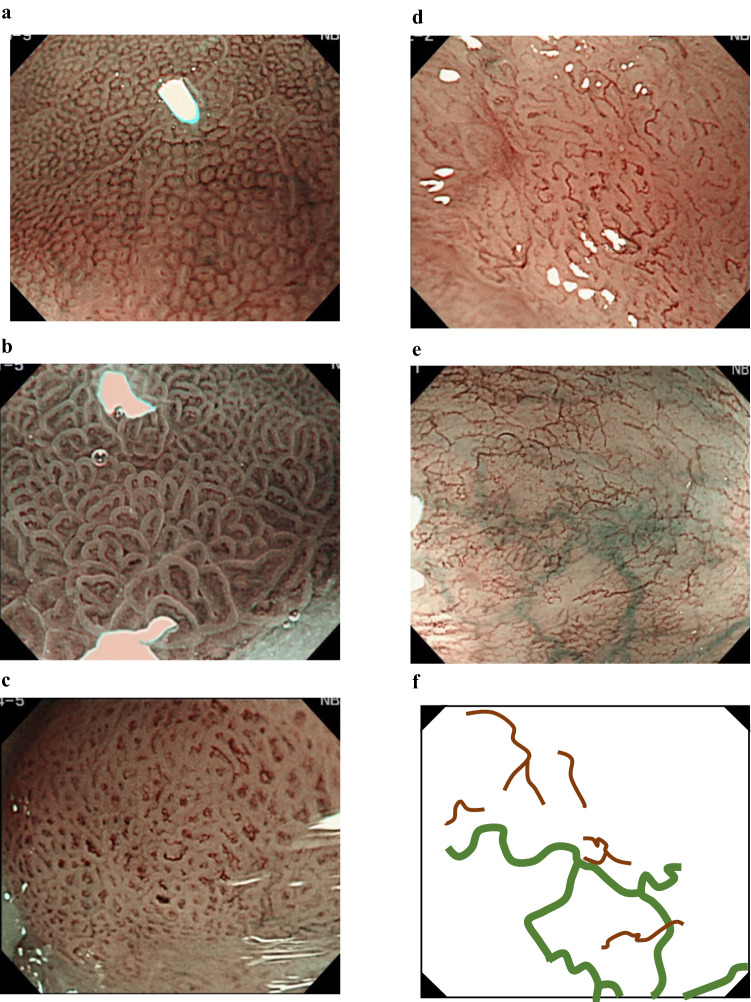
Representative endoscopic images using high-definition magnification NBI. **a** Regular mucosal pattern and regular vascular patterns. The mucosal pattern shows circular pits with similar sizes or forms arranged regularly. The vascular pattern demonstrates network-like vascular structure composed of spiral-like vessels located between pit-like mucosal patterns, and the vessel calibers change gradually. Histology from biopsy specimens showed fundic-type columnar epithelium including parietal cells and chief cells. **b** Regular mucosal pattern and regular vascular pattern. The mucosal pattern shows villous structures with density same as the surrounding area and clearly visible white zone with homogenous width. The vascular pattern located in the villous structure and the vessel calibers change gradually. Histology from biopsy showed cardiac-type columnar epithelium with specialized intestinal metaplasia and foveolar hyperplasia. **c** Irregular mucosal pattern and irregular vascular pattern. The mucosal pattern shows high-density villous patterns, and the vascular pattern demonstrates various forms with different calibers. Histology from endoscopic submucosal dissection showed well to moderately differentiated adenocarcinoma invading the lamina propria mucosae. **d** Invisible mucosal pattern and irregular vascular pattern. The vascular pattern shows irregularly bending and branching vessels. Calibers of the vessels change abruptly. Histology from endoscopic submucosal dissection showed well to moderately differentiated adenocarcinoma invading the muscularis mucosa. **e** Flat pattern with invisible mucosal pattern and regular vascular pattern. The flat pattern consists of a completely flat surface; normal-appearing, long branching vessels [brown lines in (**f**)]; and greenish thick vessels [bold green lines in (**f**)]. There is no demarcation line between completely flat area and the surrounding area. Histology of the biopsied tissue revealed tubular glands of specialized intestinal metaplasia that were covered by foveolar epithelium. **f** Schematic diagram of the endoscopic image shown in **e**

In real-time magnifying endoscopy, mucosal patterns can be visualized at a low magnification and vascular patterns at a high magnification. Based on this observation, we designed a diagnostic flowchart using the JES-BE classification (Fig. 1). First, the mucosal pattern was classified as “visible” or “invisible” and rated as “regular” or “irregular” based on the diagnostic criteria for irregularity, as reported previously [18]. The “invisible” mucosal pattern cannot be rated. Second, the vascular pattern was classified as “visible” or “invisible.” The “visible” vascular pattern included normal-appearing, long branching vessels and greenish thick vessels (GTV) previously reported and after-mentioned [16, 17]. General diagnosis was rated as “regular” or “irregular” based on mucosal plus vascular patterns. Finally, histology (“non-dysplastic” vs. “dysplastic”) was predicted according to the general diagnosis. “Dysplastic” corresponds to SBERN, including low-grade dysplasia (LGD), high-grade dysplasia (HGD), and superficial adenocarcinoma. Representative high-definition magnification NBI (HM-NBI) images are shown in Fig. 2.

General diagnosis and predicted histology were rated as follows. If mucosal and vascular patterns were both visible and had different regularity (e.g., regular mucosal pattern/irregular vascular pattern), the general diagnosis and predicted histology were “irregular” and “dysplastic,” respectively. If it was impossible to classify mucosal or vascular pattern into both regular and irregular patterns, the pattern was defined as “Unclassified” mucosal or vascular pattern. The “Unclassified” pattern was rated as “irregular” to encourage clinician to take a biopsy sample.

The mucosal surface of BE occasionally shows an invisible/absent mucosal pattern, which is known as a “flat pattern,” corresponding not to dysplastic but to non-dysplastic histology [11]. The flat pattern was originally defined as absence of pits and villi (i.e., invisible/absent mucosal pattern) with normal-appearing, long branching vessels [17]. The flat pattern mimics an absent micro-surface (mucosal) pattern, which is significantly suggestive of early gastric cancer [19, 20]. As it was difficult for clinicians working in areas with a high incidence of gastric cancer to rate the flat pattern as non-dysplastic, recent studies proposed modified criteria for the “flat pattern.” This was to enhance the diagnostic accuracy for non-dysplastic lesions [16–18]. This study used the modified criteria; according to these criteria, an invisible mucosal pattern without a distinct demarcation line and visible vascular patterns of long branching vessels [11, 15] or GTV [16–18] was rated as “regular.” Thus, a flat pattern was rated as “regular” and the predicted histology as “non-dysplastic” [11, 15–17]. Figure 2 shows representative HM-NBI images of the flat pattern according to the modified criteria.

### Patients and image collection

The study organizer (K.G.) and the working group members (J. F., R.I., M.T., A.T., Y.T., G.W., D.H., K.M., and Y.A.) retrieved still HM-NBI images from the databases of 10 participating institutions. Images of the mucosal surface of non-dysplastic and dysplastic BE were captured. The captured sites of non-dysplastic and dysplastic BE (i.e., SBERN) were all biopsied and resected endoscopically. Histology was established by a central review. The investigators who participated in the image collection were not included as reviewers in the validation (test) phase.

The study organizer and working group members obtained 277 HM-NBI images of non-dysplastic and dysplastic BE from the 10 institutions between January 2007 and December 2015. The HM-NBI images of the mucosal sites were obtained from 174 patients with BE, who underwent a targeted biopsy or endoscopic resection. The study organizer (K.G.) selected HM-NBI images that met following criteria: (1) images acquired using a high-definition magnification endoscope (GIF-H260Z; Olympus Corporation, Tokyo, Japan) and (2) images of dysplastic lesions of flat macroscopic type [23, 24]. HM-NBI images were excluded if they (1) were images of macroscopically visible lesions, such as ulcers, nodules, or plaques; (2) were considerably out-of-focus; (3) had blood or mucus attached. If more than one image was captured from a mucosal biopsy site, the highest-quality image was selected. A total of 186 HM-NBI images from 139 patients were used in this study.

The study protocol was approved by the institutional review boards of the 10 institutions including the Jikei University School of Medicine (27-008 (7892)) and this study was conducted in accordance with the modified Helsinki Declaration (1989).

### Training and validation (test) phases

#### Preparation

The HM-NBI images (*n* = 186) were stored in high-quality TIFF format, and each image was inserted into a PowerPoint (Windows 2010, Microsoft, Santa Clara, CA, USA) slide. The image slides were individually numbered. Demographic data and details of endoscopic findings, including BE lengths, presence/absence of hiatal hernia, and reflux esophagitis, were also obtained [25].

#### Image reviewer

We invited five non-experts (H.F., T.Y., Y.O., T.A., and J.F.) and five experts (T.O., K.Y., T.K., S.H., and T.S.) as reviewers. We defined experts and non-experts as having experience of magnification NBI endoscopy for over and less than 20 cases of SBERN, respectively. Diagnostic process of magnification NBI endoscopy for early gastric cancer is similar to that for SBERN [19–22], but partly different from that for SBERN, especially in flat pattern diagnosis [16, 17]. We considered that experts should be familiar with magnification endoscopy for SBERN as well as early gastric cancer. As with previous studies [16, 17], we defined experts who have experience of magnification NBI endoscopy for over 20 cases of SBERN because the experts must have the experience of magnification NBI endoscopy for over 100 cases of early gastric cancer considering the prevalence of these tumors.

None of the reviewers participated in the image collection and selection. The reviewers had no access to the clinical information, histologic data, and other imaging materials.

#### Training phase

The 10 reviewers assembled at the Jikei University Hospital. First, the study organizer (K.G.) delivered the lecture on magnification endoscopic diagnosis of non-dysplastic BE and dysplastic BE according to the JES-BE classification system. Subsequently, the 10 reviewers assessed 30 (15 non-dysplastic and 15 dysplastic histology) of the 186 HM-NBI images. The reviewers evaluated the mucosal and vascular patterns of the 30 images and discussed how to correctly predict the histology of the images. This process aimed to standardize the interpretation of HM-NBI images based on the JES-BE classification system. The 30 images in the training phase were not included in the validation (test) phase.

#### Validation (test) phase

This phase consisted of two tests. The first test was performed immediately after the training phase; the second test, at 6 weeks after the first test. The reviewers assessed 156 HM-NBI images (67 dysplastic and 89 non-dysplastic histology) in each test according to the diagnostic flowchart (Fig. 1). First, the reviewers assessed the mucosal pattern and rated it as “regular” or “irregular” based on the mucosal pattern alone (mucosal pattern diagnosis). Then, they assessed the vascular pattern and rated it as “regular” or “irregular” based on both the mucosal and vascular pattern (general diagnosis). Finally, they predicted the histology according to the diagnostic flowchart (Fig. 1). All reviewers were provided with two compact discs containing image albums for the first and second tests. The two image albums contained the same 156 HM-NBI images; however, the images were randomly arranged according to two random number tables and thus the order differed between the two albums.

### Pathologic diagnosis

Biopsied and resected specimens were stained with hematoxylin and eosin and were sent to two gastrointestinal pathologists (G.W. and Y.O.) for a central review. In the resected specimens of dysplastic lesions, the pathologists of the participating institutions put a mark on the local site of a histological preparation corresponding to each HM-NBI image; the marked specimens were evaluated in the central review. Biopsy specimens obtained from non-dysplastic mucosal sites were diagnosed as specialized intestinal metaplasia (SIM) or columnar metaplasia without SIM. The final histology established by a consensus between the two pathologists was considered the gold standard.

The degree of dysplasia was classified according to the Vienna classification of gastrointestinal epithelial neoplasia [26]. No dysplasia and indefinite for dysplasia were defined as “non-dysplastic,” and LGD, HGD, and superficial adenocarcinoma (i.e., the invasion depth is confined to the submucosa), as “dysplastic.”

In resected specimens, tumor differentiation was classified as a dominant type of differentiated or undifferentiated [27].

### Outcomes

The primary outcome measures in this study were sensitivity and specificity to the general diagnosis (mucosal plus vascular pattern) for all reviewers, including experts and non-experts. The secondary outcome measures were as follows: (1) diagnostic values, including accuracy, positive predictive value (PPV), and negative predictive value (NPV); (2) diagnostic reproducibility as evaluated by inter- and intra-observer agreements using kappa (*κ*) statistics; (3) the diagnostic values for mucosal pattern alone (mucosal pattern diagnosis) and mucosal plus vascular pattern (general diagnosis) in HM-NBI images of mucosal patterns rated as visible; and (4) overall accuracy rates based on nine combinations of mucosal and vascular patterns.

### Sample size calculation and statistical analysis

We calculated the required sample size for the validation (test) phase based on a previous study with a similar study design [15]. The previous study showed that the sensitivity and specificity of magnification NBI endoscopic classification for BE among four reviewers were 0.93 and 0.96, respectively. Accordingly, the number of images needed in our study, which includes 10 reviewers, was calculated based on the following: (1) expected sensitivity and specificity of 0.85 (threshold value, 0.80) and 0.90 (threshold value, 0.85), respectively; such values are attributed to the larger number of non-expert reviewers in our study than in the previous study, which may in turn lower both sensitivity and specificity; nevertheless, the expected sensitivity and specificity values are acceptable in practical endoscopy; (2) one-sided type 1 error rate of 0.05 after multiplicity adjustment; and (3) overall power = 0.95 (1.0—overall type 2 error rate). The required sample size was 154 HM-NBI images, including 88 dysplastic and 66 non-dysplastic lesions. Of the 186 HM-NBI images, 156 images were selected for the validation phase (89 dysplastic and 67 non-dysplastic lesions).

Diagnostic values for the prediction of dysplastic histology were calculated. The degree of coincidence was calculated using *κ*-statistics. Inter-observer agreements were calculated in the first test and intra-observer agreements between the first and second tests. The *κ* values were interpreted based on the following standards for strength of agreement developed: poor (*κ* < 0), slight (0 ≤ *κ* ≤ 0.20), fair (0.21 ≤ *κ* ≤ 0.40), moderate (0.41 ≤ *κ* ≤ 0.60), substantial (0.61 ≤ *κ* ≤ 0.80), and almost perfect (0.81 ≤ *κ* < 1.0) [28]. Substantial and almost perfect strengths were regarded as a good agreement with high reliability. In all analyses, the level of statistical significance was set at α = 0.05. Statistical analyses were performed using SAS software (version 9.4, SAS Institute, Cary, NC).

## Results

Patient demographics and lesion characteristics are listed in Table 1.

**Table 1:** Patient demographics and lesion characteristics

Age, median (IQR), years	66 (56–77)
Sex, male/female, *n*	97/19
BE length	
Short segment/long segment, *n*	88/28
Circumferential/maximal extent, median (IQR), cm	1.0 (0.4–2.0)/2.3 (1.2–5.0)
Hiatal hernia, *n* (%)	59 (66)
Reflux esophagitis, *n* (%)	84 (72)
Grade M/A/B/C/D	61/7/14/2/0
Histology associated with NBI magnification images	
Non-dysplastic, *n* (CM/SIM)	67 (37/30)
Dysplastic, *n* (LGD/HGD/superficial adenocarcinoma)	89 (18/41/30)
Adenocarcinoma (depth), M/SM	29/1
Adenocarcinoma (grading), differentiated/undifferentiated	27/3

*QR* interquartile range, *BE* Barrett’s esophagus, *CM* columnar metaplasia without specialized intestinal metaplasia, *SIM* specialized intestinal metaplasia, *LGD* low-grade dysplasia, *HGD* high-grade dysplasia; *M* mucosal layer, *SM* submucosal layer

Table 2 lists the diagnostic values and observer agreement for predicting dysplastic histology in the first test. The sensitivity and specificity to the general diagnosis for all reviewers were 87% and 97%, respectively. These values exceeded the threshold values based on the sample size calculation. Overall accuracy, PPV, and NPV were 91, 98, and 83%, respectively. No significant difference in any diagnostic values between the experts and non-experts was found. The κ values of diagnostic reproducibility for all reviewers were at least substantial. The strengths of inter-observer and intra-observer agreements for the prediction of dysplastic histology were substantial (*κ* = 0.77) and almost perfect (*κ* = 0.83), respectively. No significant difference in inter-observer and intra-observer agreements between experts and non-experts was found.

**Table 2:** Diagnostic values and observer agreement for predicting the histology of superficial Barrett’s esophagus-related neoplasms

General diagnosis^a^ (*n*, reviewers’ assessments)	Sensitivity (95% CI)	Specificity (95% CI)	Accuracy (95% CI)	PPV (95% CI)	NPV (95% CI)	Inter-observer agreements, κ value (95% CI)	Intra-observer agreements, κ value (95% CI)
Overall (*n* = 1560)	87 (85–89)	97 (96–99)	91 (90–93)	98 (97–99)	83 (81–86)	0.77 (0.75–0.80)	0.83 (0.80–0.86)
Experts (*n* = 780)	87 (84–90)	98 (96–99)	91 (89–93)	98 (97–99)	83 (80–86)	0.75 (0.70–0.80)	0.83 (0.79–0.87)
Non-experts (*n* = 780)	87 (84–91)	97 (96–99)	91 (89–93)	98 (96–99)	84 (80–87)	0.79 (0.74–0.84)	0.83 (0.80–0.87)

*PPV* positive predictive value, *NPV* negative predictive value, *CI* confidence interval

^a^General diagnosis and predicted histology based on mucosal plus vascular pattern

Table 3 lists the diagnostic values for mucosal pattern alone (mucosal pattern diagnosis) and mucosal plus vascular pattern (general diagnosis) among the 956 reviewers’ assessments of “visible” mucosal pattern. No significant difference was noted in any of the diagnostic values between mucosal pattern alone and mucosal plus vascular pattern.

**Table 3:** Diagnostic values for mucosal pattern alone (mucosal pattern diagnosis) and mucosal plus vascular pattern (general diagnosis) among 956 reviewers’ assessments of mucosal patterns rated as “visible”

	Sensitivity (95% CI)	Specificity (95% CI)	Accuracy (95% CI)	PPV (95% CI)	NPV (95% CI)
Visible MP alone (*n* = 956)	82 (78–86)	98 (97–99)	88 (86–91)	98 (97–99)	78 (75–82)
Visible MP plus VP (*n* = 956)	81 (78–85)	98 (97–99)	88 (86–90)	98 (97–99)	78 (74–81)

*MP* mucosal pattern, *VP* vascular pattern, *PPV* positive predictive value, *NPV* negative predictive value, *CI* confidence interval

Table 4 lists the number of reviewer assessments and the overall accuracy of predicting histology based on nine combinations of mucosal and vascular patterns. Both mucosal and vascular patterns were visible and graded identically (e.g., regular mucosal pattern/regular vascular pattern) in 47.6% of the reviewer assessments. All combinations with regular patterns (446 assessments) and irregular patterns (296 assessments) predicted non-dysplastic and dysplastic BE with high overall accuracy values (90.6% and 97.0%, respectively). The combination of invisible mucosal pattern and irregular vascular pattern had the highest incidence (518 assessments, 33.2%) and second highest overall accuracy value (95.4%) for the prediction of dysplastic histology. The overall accuracy of invisible mucosal pattern was 86.4% (including regular, irregular, and invisible vascular patterns).

**Table 4:** Overall accuracy for predicting the histology of Barrett’s esophagus in all combinations of mucosal and vascular patterns

Mucosal pattern	Vascular pattern	Reviewer assessments of each combination	Reviewer assessments of general diagnosis^a^	Histology	Overall accuracy
		*n* (%)	Dysplastic, *n*, (%)	Dysplastic, *n*, (%)	% (95% CI)
Regular	Regular	446 (28.6)	3 (0.7)	43 (9.6)	90.6 (87.5–93.1)
	Irregular	50 (3.2)	38 (24)	21 (42.0)	78.0 (64.0–88.5)
	Invisible	118 (7.6)	0 (0)	12 (10.2)	89.8 (82.9–94.6)
Irregular	Regular	24 (1.5)	17 (70.8)	17 (70.8)	50.0 (29.1–70.9)
	Irregular	296 (19.0)	288 (97.3)	291 (98.3)	97.0 (94.3–98.6)
	Invisible	22 (1.4)	21 (95.5)	22 (100)	95.5 (77.2–99.9)
Invisible	Regular	80 (5.1)	0 (0)	18 (22.5)	77.5 (66.8–86.1)
	Irregular	518 (33.2)	448 (86.5)	460 (88.8)	95.4 (93.2–97.0)
	Invisible	6 (0.4)	4 (66.7)	6 (100)	66.7 (22.3–95.7)

*CI* confidence interval

^a^General diagnosis and prediction based on mucosal plus vascular pattern

## Discussion

We developed the JES-BE classification system, including a diagnostic flowchart (Fig. 1), for the diagnosis of SBERN and conducted this nationwide multicenter study to evaluate its diagnostic validity and reliability. We found high values of diagnostic accuracy, including sensitivity/specificity, and observer agreement among 10 endoscopists. No significant difference in any values of diagnostic accuracy and observer agreement between experts and non-experts was found.

We developed the JES-BE classification system, including the diagnostic flowchart (Fig. 1), based on practical magnification endoscopic procedure. The results of high diagnostic values and good observer agreement may prove the diagnostic utility of the JES-BE classification system and the diagnostic flowchart. Although many classifications of magnification endoscopy for predicting dysplastic BE have been developed, none of them have included a diagnostic flowchart, which we believe is particularly helpful to non-expert users.

The JES-BE classification system was developed to be simple, easy to understand, and widely available. To achieve this, it was important to simplify both the diagnostic criteria and the diagnostic process. To this end, as previously described [18], we clearly and precisely specified the JES-BE criteria for easy understanding and estimation of mucosal/vascular patterns (regular or irregular) even among non-experts. Most studies related to magnification endoscopic classifications did not include non-experts [11–15]; in our study, we included non-experts and found high values of diagnostic accuracy and observer agreement among non-experts and experts. Hence, the JES-BE classification may be an acceptable and reliable even among non-experts and thus may have the potential for a wide application.

**Fig. 3:**
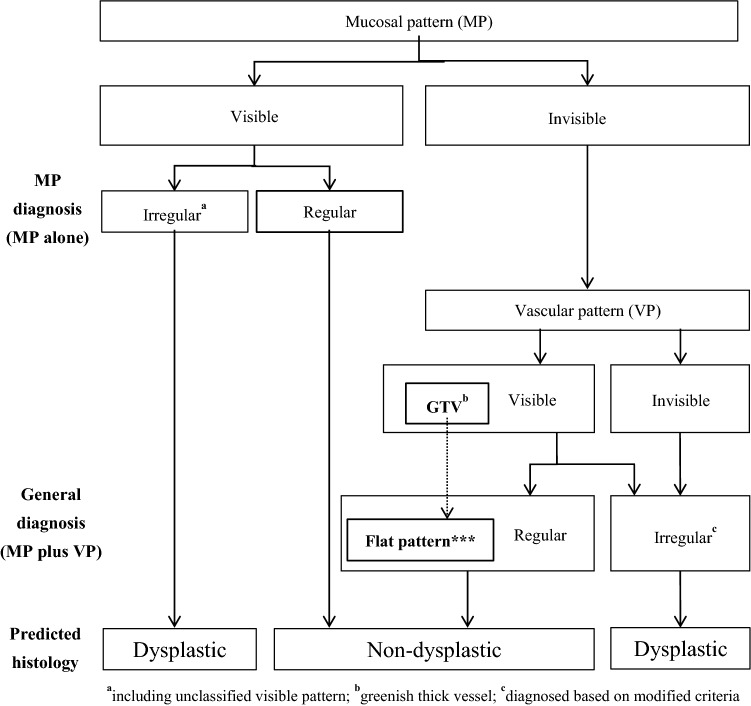
Diagnostic flowchart of simplified the JES-BE classification system

We investigated the number of reviewer assessments and the overall accuracy of predicting histology in all combinations of mucosal and vascular patterns. In nearly half of reviewer assessments, both mucosal and vascular patterns were visible and graded the same (e.g., regular mucosal and regular vascular patterns). All combinations of regular mucosal and vascular patterns and of irregular mucosal and vascular patterns predicted non-dysplastic and dysplastic histology with high overall accuracy values (90.6% and 97.0%, respectively). These results support the relevance of the JES-BE criteria for regular and irregular patterns in the accurate prediction of histology. Further, we found that among the assessments of mucosal pattern rated as “visible,” there were no significant differences in any of the diagnostic values between mucosal pattern alone and mucosal plus vascular pattern. In other words, the additional assessment of vascular pattern did not improve diagnostic accuracy when the mucosal pattern was rated as “visible.” Thus, we propose to simplify the diagnostic process by omitting assessments of vascular patterns and mucosal pattern alone (Fig. 3).

We suggested that invisible mucosal and irregular vascular patterns would be a key combination to predict dysplastic histology because such a combination accounted for the highest proportion of the reviewer assessments and showed remarkably high overall accuracy for dysplastic histology (95.4%). Invisible mucosal patterns correspond to absent micro-surface patterns in magnification endoscopy classification for early gastric cancer [19]. The combination of invisible/absent mucosal pattern and irregular vascular pattern is suggestive of dysplastic or cancerous lesions in BE and the stomach.

However, occasionally, the mucosal surface of BE shows an invisible/absent mucosal pattern corresponding not to dysplastic but to non-dysplastic histology, which is known as a “flat pattern” [11]. The flat pattern was originally defined as invisible/absent mucosal pattern with normal-appearing, long branching vessels [17]. Moreover, the flat pattern mimics an absent micro-surface (mucosal) pattern, which is significantly suggestive of early gastric cancer [19, 20]. A previous study demonstrated that a flat pattern makes the prediction of a non-dysplastic BE histology extremely difficult; the prediction accuracy among non-experts was only 13% [17]. To overcome this, modified criteria for flat pattern were proposed, in which no clear demarcation line and a GTV were added. Using the modified criteria, previous studies showed significantly positive results [16, 17]. We thus incorporated the modified criteria for flat pattern into the JES-BE criteria. The modified criteria for flat pattern potentially contributed to the high accuracy values of invisible mucosal patterns especially for non-experts in this study.

Recently, Barrett’s International NBI Group (BING) proposed simplified criteria for magnification endoscopic diagnosis of dysplasia in BE. The BING study demonstrated high values of diagnostic accuracy (sensitivity 80.4% and specificity 88.4%) and inter-observer agreement (*κ* = 0.681, substantial) for the prediction of dysplasia [14]. While our study showed higher diagnostic values, except for NPV, than the BING study, a simple comparison between the two is impossible because the study methods were different and diagnostic accuracy values, other than sensitivity and specificity, were affected by the prevalence rate (i.e., the proportion of dysplastic lesions).

More recently, artificial intelligence (AI) has been applied for the detection of BERN [29]. The latest meta-analysis showed that AI yielded significantly higher diagnostic performance (sensitivity 88.0%, specificity 90.4%) than human endoscopists. AI may have a potential to improve the diagnostic performance of JES-BE classification. Thus, a new AI diagnostic system based on the JES-BE classification should be developed by learning mucosal patterns at a low magnification and vascular patterns at a high magnification according to our proposed simplified diagnostic flowchart (Fig. 3). We considered that the developed AI may provide an ideal diagnostic system for identifying SBERN.

This study has some limitations. First, this is a retrospective study that is based on a review of selected images. Selection bias must be considered because only selected high-quality images were used in the validation (test) phase. However, this process of selection could not be avoided considering the results of the sample size calculation. Although we should have selected the images randomly to minimize bias, we believe that using the best images was vital because the reviewers did not have the advantage of real-time viewing. Second, all of the non-experts who participated in this study were working at a high-volume center or academic center. The non-experts thus have substantial diagnostic ability using magnification endoscopy for other gastrointestinal cancer, including early gastric cancer; nevertheless, we recruited non-experts whose experience in magnification endoscopy involved < 20 SBERN cases. The absence of a significant difference in diagnostic abilities between the non-experts and experts could be attributed to the former’s substantial experience in magnification endoscopy. Third, most Western studies on magnification endoscopy criteria for diagnosing dysplasia in BE separated LGD and HGD/superficial adenocarcinoma [15, 30, 31]. In our study, we investigated the diagnostic performance of the JES-BE criteria for dysplasia, including LGD. This discrepancy could be attributed to the difference in histologic criteria and treatment strategy between Japan and Western countries [32]. LGD diagnosed by Western pathologists corresponds to a differentiated adenocarcinoma with low-grade atypia diagnosed by Japanese pathologists. Similar to HGD/mucosal adenocarcinoma, LGD is usually an indication for endoscopic resection in Japan [5]. Fourth, in this study, there was no significant difference in any of the diagnostic values between mucosal pattern alone and mucosal plus vascular pattern. However, there is potential information bias in this finding. The reviewers could see both the mucosal and vascular patterns in their first assessment of mucosal pattern, because the HM-NBI images used in this study were highly magnified images. Therefore, further studies using endoscopic images at a low magnification, i.e., where only a mucosal pattern is discernible, are warranted.

## Conclusion

In summary, we conducted a nationwide multicenter study to validate the diagnostic utility of the JES-BE classification system in SBERN. This study showed that the JES-BE criteria for predicting dysplastic histology have high diagnostic accuracy and reproducibility based on the evaluation of both non-experts and experts. Moreover, the results suggested that the JES-BE criteria are acceptable and reliable, regardless of the clinician’s experience level, and have the potential to be widely used.
